# Identifying Predictors of University Students’ Wellbeing during the COVID-19 Pandemic—A Data-Driven Approach

**DOI:** 10.3390/ijerph18136730

**Published:** 2021-06-22

**Authors:** Chang Liu, Melinda McCabe, Andrew Dawson, Chad Cyrzon, Shruthi Shankar, Nardin Gerges, Sebastian Kellett-Renzella, Yann Chye, Kim Cornish

**Affiliations:** School of Psychological Sciences, Turner Institute for Brain and Mental Health, Monash University, Clayton, VIC 3800, Australia; chang.liu5@monash.edu (C.L.); Melinda.McCabe@monash.edu (M.M.); Andrew.Dawson2@monash.edu (A.D.); Chad.Cyrzon@monash.edu (C.C.); Shruthi.Shankar@monash.edu (S.S.); Nardin.Gerges@monash.edu (N.G.); skel0011@student.monash.edu (S.K.-R.); yannying.chye@monash.edu (Y.C.)

**Keywords:** COVID-19, university students, psychological wellbeing, machine learning, intervention

## Abstract

Background: The COVID-19 pandemic has posed risks to public mental health worldwide. University students, who are already recognised as a vulnerable population, are at elevated risk of mental health issues given COVID-19-related disruptions to higher education. To assist universities in effectively allocating resources to the launch of targeted, population-level interventions, the current study aimed to uncover predictors of university students’ psychological wellbeing during the pandemic via a data-driven approach. Methods: Data were collected from 3973 Australian university students ((median age = 22, aged from 18 to 79); 70.6% female)) at five time points during 2020. Feature selection was conducted via least absolute shrinkage and selection operator (LASSO) to identify predictors from a comprehensive set of variables. Selected variables were then entered into an ordinary least squares (OLS) model to compare coefficients and assess statistical significance. Results: Six negative predictors of university students’ psychological wellbeing emerged: White/European ethnicity, restriction stress, perceived worry on mental health, dietary changes, perceived sufficiency of distancing communication, and social isolation. Physical health status, emotional support, and resilience were positively associated with students’ psychological wellbeing. Social isolation has the largest effect on students’ psychological wellbeing. Notably, age, gender, international status, and educational level did not emerge as predictors of wellbeing. Conclusion: To cost-effectively support student wellbeing through 2021 and beyond, universities should consider investing in internet- and tele- based interventions explicitly targeting perceived social isolation among students. Course-based online forums as well as internet- and tele-based logotherapy may be promising candidates for improving students’ psychological wellbeing.

## 1. Introduction

The coronavirus (COVID-19) pandemic resulted in over 79.2 million confirmed cases and 1.7 million deaths globally by 29 December 2020 [1]. Both the pandemic and the measures taken to control its spread have caused a range of negative personal, social, and economic consequences for the general population, including unemployment, economic uncertainty, anxiety, social isolation, and increased sedentary behaviours [2,3,4,5]. There was also a significant increase in the national prevalence of clinical levels of distress and subjective mental health concerns at the early stage of lockdown (April 2020) compared with data from previous years (2018–2019) [6].

Compared with the general population, university students may be more vulnerable to mental health issues stemming from the pandemic. Much international research has identified university students as a high-risk group for mental health conditions [7,8,9], especially at critical transition points of their education [10]. During COVID-19, university students faced unique stressors beyond the common health, economic, and social stressors that impacted almost everyone. Higher education was severely disrupted as courses moved online (with varying degrees of success), campus life grinded to a halt, formative personal and intellectual experiences were snatched away, and students grew increasingly despondent about the impacts of the pandemic on their future career options in a crippled world economy. Unsurprisingly, university students exhibited significantly higher rates of depression (38.6% vs. 15.8%) and anxiety (21.5% vs. 8.8%) symptoms compared with the general population during the pandemic [11].

A host of factors have been implicated in understanding elevated psychopathology in university students in COVID-19: gender, age, ethnicity, cultural background (English speaking or non-English speaking), family income, educational level, academic dissatisfaction, anxiety, worry, and sense of coherence [11].

In much of this work, the relative importance of these variables has not been established. While each variable might be revealed to be significant and important in its own right across individual studies, we are unaware of any study that has concurrently measured all these variables and then objectively determined their relative importance to university students’ mental health or wellbeing (i.e., ‘played the variables off against each other’). For institutions required to make quick decisions about which particular population-level interventions to launch initially in response to the pandemic—and which particular at-risk populations to target (e.g., international students, graduate students)—such information would be extremely valuable. This study aims to provide it in the Australian context.

The present study furthers extends on past work by (a) focusing on students’ everyday wellbeing instead of mental health or psychopathology; (b) going beyond the early stages of lockdown in gathering data; and (c) avoiding some of the limitations of traditional statistical models in the exploratory stage (e.g., type I errors, model overfitting, and multicollinearity) [12,13]. We expect the regularized feature selection (i.e., LASSO) method we employ to reveal a set of critical variables that shares some overlap with variables identified in past work, but also identifies novel intervention targets.

## 2. Method

### 2.1. Participants and Procedure

Data were collected at five time points across 2020 (i.e., May, July, August, October, and December). At each time point, invitation links to an online survey were sent to undergraduate and postgraduate students enrolled with Monash University (i.e., a major Australian public research university) at either one of its Australian or Malaysian campuses. Only participants aged 18 and above who provided informed consent were eligible for participation. The current study consisted of 3973 participants across all time points. The overall response rate was 4.7% (out of 84,284 students enrolled in 2020). Specifically, 1689 students participated in May, 940 students participated in July, 595 students participated in August, 407 students participated in October, and 342 students participated in December. A subsample of the total sample (students living in residential halls) were offered an opportunity to win a digital gift card reimbursement for their participation as they completed a tailored version of the survey, with additional measures not reported here. The rest of the sample received no reimbursement. The study followed the Helsinki Declaration and was approved by the Human Research Ethics Committee at Monash University (Project Number 23969).

### 2.2. Measures

All data were collected online via Qualtrics. Demographic information including age, gender, ethnicity, degree, living conditions, and enrollment status (i.e., enrolled campus and identity as domestic/international students) was collected at the beginning of the survey. Measures of depression, anxiety, sleep, and perceived stress were taken, but not included in the current study owing to their high conceptual overlap with the outcome measure of interest, psychological wellbeing. The following measures were thus administered in the current study.

Brief Resilience Scale [14]: This is a six-item scale measuring resilience (i.e., the ability to bounce back under stressful situations) [14]. Sample items include ‘I tend to bounce back quickly after hard times’ and ‘It does not take me long to recover from a stressful event’. Responses range from 1 (Strongly Disagree) to 5 (Strongly Agree). The total score was the measure of interest for our study. The scale has been validated cross-culturally among different populations, with consistent reliability (Cronbach’s alpha ranging from 0.80 to 0.90) [15].

Patient-Reported Outcomes Measurement Information System (PROMIS) Emotional Support Scale [16]: This is a four-item scale measuring perceived emotional support (i.e., individuals’ perception of psychological resources they may receive from their social network when needed) [17]. Sample items include ‘I have someone who will listen to me when I need to talk’ and ‘I have someone to talk with when I have a bad day’. Responses range from 1 (never) to 5 (always). The T-score was the measure of interest for our study. The T-score was calculated by rescaling the total score into a standardized score with a mean of 50. The PROMIS Emotional Support scale has been validated among young adults, with excellent reliability (Cronbach’s alpha = 0.95) [18].

Patient-Reported Outcomes Measurement Information System (PROMIS) Social Isolation Scale [16]: This is a four-item scale measuring perceived isolation from others. Sample items include ‘I feel left out’ and ‘I feel isolated from others’. Responses range from 1 (never) to 5 (always). The T-score was the measure of interest for our study. The PROMIS social isolation scale has been validated among young adults, with excellent reliability (Cronbach’s alpha = 0.92) [19].

The five-item World Health Organization Well-Being Index (WHO-5) [20]: This is a widely used scale measuring psychological wellbeing. The scale contains items on depression (negatively phrased, ‘I have felt cheerful in good spirits’), fatigue (negatively phrased, ‘I woke up feeling fresh and rested’), and anxiety and stress (negatively phrased, ‘I have felt calm and relaxed’). The scale has been used as a proxy measure of clinical depression [20]. Responses ranged from 0 (At no time) to 5 (All of the time). The raw score was multiplied by four to get the final score. Individuals who scored 50 and below (i.e., raw scores below 13) were considered as demonstrating poor wellbeing [20]. The cut-off has been used as an index of poor wellbeing among university students [21,22]. The scale has been validated cross-culturally in both clinical and general population, with consistent reliability (Cronbach’s alpha ranging from 0.88 to 0.93) [23,24,25].

COVID-Specific Items: These items developed in-house (see Table S2: Summary of COVID-Specific Items) covered perceived negative impacts from the pandemic in the preceding two weeks. Each item is rated on a five-point Likert scale ranging from 0 to 4. Higher scores indicated more perceived negative impacts stemming from the pandemic.

Lifestyle Factors: All relevant items were created for the purposes of this study. Three lifestyle factors were examined: time spent outside, perceived sufficiency of using distance-communications, and dietary change. Time spent outside items examined how many days participants have spent outside in the last two weeks (ranging from ‘No day’ to ‘Everyday’). Perceived sufficiency of using distance-communications items examined how participants perceived using distance-communication during the pandemic (ranging from ‘Not at all’ to ‘Extremely’). Dietary change items examined whether participants’ diets have been worsened during the pandemic.

Physical Health Status: A single item question, ‘How do you rate your current physical health status, was used to examine participants’ self-rated physical health status. The responses ranged from ‘Poor’ to ‘Excellent’.

### 2.3. Analyses

LASSO regression was used for unbiased selection of predictors for university students’ psychological wellbeing during COVID-19. LASSO is suitable for analysing high-dimensional data and has been proven to provide more stable results as well as identify fewer noise predictors compared with commonly-used variable selection approaches [26,27]. Additionally, LASSO overcomes the concern of multicollinearity by introducing a regularization parameter (λ) that will, in effect, shrink the coefficients of irrelevant predictors to zero (for details, see [27]). There is an emerging trend of using LASSO for variable selection in the psychology where explanatory variables are often correlated [28,29,30].

For the current study, we adopted the approach described by Strohmeier and colleagues [30] for feature selection and follow-up model fitting. Briefly, all predicting variables were entered into the LASSO model for feature selection (for the full list of predicting variables, see Table 1: Participants Characteristics). The data were randomly split (80/20) into training and test sets to avoid overfitting. The model was initially trained on the training set and then evaluated on the test set to assess out of sample performance. In order to compare coefficients and assess statistical significance and effect sizes, the selected variables (i.e., coefficients not shrunk to zero) were entered into an OLS model. LASSO regression was conducted via Python ‘sklearn’ package. OLS regression was conducted via SPSS version 27.

## 3. Results

The median age of participants was 22 (range = 18 to 79, SD = 8.2) and 70.6% identified as female. The prevalence of poor wellbeing (scored ≤50 on WHO-5) was 66.3% among participants, with 33.1% indicating potential risk of clinical depression (i.e., scored ≤28 on WHO-5). Detailed participants characteristics are presented in Table 1.

Among all variables entered into the LASSO model, nine variables were identified as prominent predictors of psychological wellbeing. (i.e., coefficients not shrunk to zero), namely, White/European ethnicity, physical health status, restriction stress, worry about COVID-19 impact on mental health, dietary changes, perceived sufficiency of distancing communication, social isolation, resilience, and emotional support (Table S1: LASSO Model for Psychological Wellbeing). These predictors were further entered into an OLS model (Table 2).

The results from the OLS model showed that White/European ethnicity (B = −0.10, *p* < 0.01), restriction stress (B = −0.06, *p* < 0.01), perceived worry on mental health (B = −0.13, *p* < 0.01), dietary changes (B = −0.10, *p* < 0.01), perceived sufficiency of distancing communication (B = −0.05, *p* < 0.01), and social isolation (B = −0.23, *p* < 0.01) were negatively associated with students’ psychological wellbeing during the pandemic. In contrast, physical health status (B = 0.22, *p* < 0.01), resilience (B = 0.17, *p* < 0.01), and emotional support (B = 0.12, *p* < 0.01) were positively associated with students’ psychological wellbeing during the pandemic. Based on Cohen’s guidelines, f2 ≥ 0.02, f2 ≥ 0.15, and f2 ≥ 0.35 refer to small, medium, and large effect sizes, respectively [31]. In the current study, perceived social isolation has a large effect on students’ psychological wellbeing (Cohen’s f2 = 0.39). Meanwhile, all other predictors have small effects on students’ psychological wellbeing.

## 4. Discussion

Promoting students’ psychological wellbeing during the pandemic has been a priority of higher education institutions. Developing and implementing effective mental health strategies at a population level require higher education providers to understand priority areas for interventions. To address this, the current study utilized the techniques from machine learning (i.e., feature selection and train-test validation) and regularized regression (i.e., LASSO) to explore prominent predictors of students’ psychological wellbeing during the COVID-19 pandemic (from May to December 2020). Six negative predictors of university students’ psychological wellbeing were identified, namely, White/European ethnicity, restriction stress, perceived worry on mental health, dietary changes, perceived sufficiency of distancing communication, and social isolation. In contrast, physical health status, emotional support, and resilience positively predict students’ psychological wellbeing.

To allocate resources appropriately, it is important for university administrators to understand the magnitude of the effect of each of the significant predictors. Effect sizes have been used as a measure of practical significance to complement the significance testing (i.e., *p*-values) and are encouraged to be reported by the statistical community [32]. In the current study, we found that lifestyle changes, COVID-related concerns, physical health status, and protective psychological factors (i.e., resilience and emotional support) were significant predictors of students’ wellbeing, yet with small effect sizes.

Among the pool of significant predictors, only social isolation has a large effect size on students’ wellbeing during the pandemic. This aligns with previous research that reported perceived isolation and loneliness played a key role in elevated psychopathology in COVID-19 [33,34]. During the pandemic, students faced challenges including government-enforced physical distancing, travel bans, and campus closure throughout the year, likely resulting in perceived isolation. Although the move to online learning may have created a valuable alternative to help students continue their education remotely, it has been found that learning online produces an increased sense of loneliness and isolation compared with attending face-to-face classes [35]. The physical proximity in face-to-face classes provides a natural channel for students to make connections and develop social networks [35,36]. The disruption of interpersonal connection and relationship building derived from restriction regulations was likely to increase students’ feeling of isolation and further contribute to poor psychological wellbeing during the pandemic.

In the current sample, we found 66.3% of students reported poor wellbeing, with 31.1% with wellbeing scores so low they may indicate risk of clinical depression (i.e., scored ≤28 on WHO-5). The high rates of poor wellbeing among students indicated that there may be an increased need for mental health services. Even pre-pandemic, it has been reported that the capacity of Australian universities’ counselling services may not meet students’ needs, resulting in long waiting periods and limited numbers of sessions [37]. Further, the revenue shortfall in higher education sectors caused by closed borders and reductions in international student enrolments may lead to a reduction in mental health budgets and, therefore, capacity. Altogether, it will be critical for university administrators to allocate the limited resources available to areas of the highest priority when addressing students’ wellbeing.

Based on our results, social isolation should be prioritized when developing and implementing wellbeing promotion interventions. Internet- and tele-based interventions have received increasing attention owing to the physical distancing regulations [38,39]. When targeting social isolation, Thai and colleagues [40] found that students with official course Facebook groups reported higher levels of social connectedness, better relationship with faculties, and less course related stress compared with those without them. Implementing course-based forums by institutions may be a feasible approach to reduce perceived isolation as well as help students stay connected with their peers and instructors during the pandemic. Additionally, a systematic review on interventions to reduce social isolation during COVID-19 proposed that logotherapy, which focuses on meaning-making, may provide promising results for reducing perceived isolation [41]. Internet- and tele-based logotherapy may be another promising candidate for improving students’ wellbeing.

Previous research suggested that well-established risk factors, including age [42], gender [43], and educational level [22], may continue to impacting students’ mental health during the pandemic. Additionally, it has been proposed that international students, who have been identified as a vulnerable group pre-pandemic, would be at heightened risk of poor mental health during COVID-19 [44,45]. Yet, these variables were not identified as significant predictors by LASSO in the current study. Interestingly, we found that White/European ethnicity was a significant negative predictor of psychological wellbeing. Further research is required to explore whether this may be due to differences in thinking styles between the East and West. Our results suggested that the COVID-19 pandemic may present unprecedented stressors to students. To complement previous research, future studies would benefit from utilizing exploratory approaches, with a reduced reliance on ‘well-established’ risk factors that were identified pre-pandemic.

One key strength of the current study is employing a large set of potential predictors and objectively determining their relative importance to university students’ wellbeing. The utilization of LASSO provides an unbiased way for all potential variables to play against each other. Despite its strengths, several limitations should be noted for the current study. Firstly, owing to its cross-sectional design, we were not able to draw any causal conclusions. Secondly, the majority of the current sample were females (70.6%), which may have an impact on participants’ self-reported mental health symptoms [46]. Thirdly, each lifestyle factor in the current study was assessed via a single item. Future studies would benefit from replicating the current results with validated scales assessing lifestyle factors. Fourthly, the current results should be interpreted with caution owing to the limited numbers of participants from the Malaysian campus. Fifthly, participants’ physical health status was measured by a single item and should be interpreted with caution. It should be noted that the current study comprised a large set of variables, which may have a trade-off between comprehensiveness and conciseness. Future research is needed to examine current findings with validated health status assessments. Lastly, despite aligning with the response rate from previous research using an online recruitment approach (e.g., 2.2–4.7%, [47]), the response rate was relatively low for the current study, perhaps due to lack of incentive given the time taken for participants to complete it. Nonetheless, the sample size was larger than other comparable studies (e.g., [22,43]) with more timepoints included.

## 5. Conclusions

The COVID-19 pandemic has posed substantive and long-term risks to public mental health worldwide. Ongoing research throughout 2020 and into 2021 clearly demonstrates that university students are at heightened risk for developing serious mental health issues. This, together with revenue shortfalls, represents inevitable challenges on already overloaded university mental health systems. Future research should prioritize identifying at-risk populations, providing solutions that may support students’ needs within limited resources as well as exploring potential prevention and early interventions that may benefit at population-level. Further, owing to the potential for ongoing economic impacts caused by COVID-19 restriction measures, as well as prolonged physical-distancing requirements and a rise in remote working, future studies could benefit in examining the efficiency of cost-effective resources and interventions that may be delivered online or through telehealth services. The pandemic nevertheless offers the opportunity for stakeholders to update the current university mental health system in preparation for emergency situations and leverage resources on developing online resources that are accessible to all students in need. Through a data-driven approach, we found that social isolation has the largest effect on students’ well-being and should be targeted for intervention. Course-based online forums as well as internet- and tele-based logotherapy may be promising candidates for improving students’ psychological wellbeing.

## Figures and Tables

**Table 1:** Participants characteristics.

Variable	
Age, *Median (SD)*	22 (8.2)
Female Sex, *N* (%)	2805 (70.6)
International Students, *N* (%)	1318 (33.2)
Enrolled in Australia, *N* (%)	3320 (83.6)
Level of Education, *N* (%)	
Undergraduate	1664 (41.9)
Undergraduate with Double Degree	325 (8.2)
Honour	645 (16.2)
Postgraduate	1339 (33.7)
Ethnicity, *N* (%)	
East Asian	739 (18.6)
South Asian	428 (10.8)
Southeast Asian	827 (20.8)
White/European	1626 (40.9)
Other	353 (8.9)
Living Conditions, *N* (%)	
Live Alone	732 (18.4)
Live on campus	244 (6.1)
Time Point, *N* (%)	
May	1689 (42.5)
July	940 (23.7)
August	595 (15.0)
October	407 (10.2)
December	342 (8.6)
Physical Health Status, *M (SD)*	2.3 (1.1)
COVID-related Items, *M (SD)*	
Worry about being infected	1.5 (1.1)
Worry about friends or family being infected	1.9 (1.2)
Worry about physical health being influenced by Coronavirus	1.5 (1.2)
Worry about mental health being influenced by Coronavirus	2.2 (1.3)
Worry about having enough money and resources	2.0 (1.4)
Worry about staying safe when leaving the house	2.0 (1.2)
Restriction Stress	1.7 (1.2)
Lifestyle Factors	
Time spent outside during last two weeks, *N* (%)	
No days	1914 (48.2)
1–2 days per week	802 (20.2)
3–4 days per week	376 (9.5)
5–6 days per week	352 (8.9)
Everyday	529 (13.3)
Diet Worsened, *N* (%)	2040 (51.3)
Perceived Sufficiency of Distance Communication, *M (SD)*	1.9 (1.2)
Psychological Factors, *M (SD)*	
Emotional Support	51.0 (9.3)
Social Isolation	54.1 (9.1)
Resilience	18.6 (4.6)
Psychological Wellbeing	42.0 (21.2)
Psychological Wellbeing ≤ 50, *N* (%)	2636 (66.3)
Psychological Wellbeing ≤ 28, *N* (%)	1235 (31.1)

Note: All variables mentioned above were entered into the LASSO model for feature selection.

**Table 2:** OLS model for psychological wellbeing.

Predictors	*B*	*SE*	*p*	Cohen’s f2
Distance Communication Sufficiency	−0.05	0.22	<0.01	<0.01
Emotional Support	0.12	0.03	<0.01	0.01
Dietary Change	−0.10	0.53	<0.01	0.01
Physical Health	0.22	0.25	<0.01	0.10
Resilience	0.17	0.06	<0.01	0.03
Restriction Stress	−0.06	0.24	<0.01	<0.01
Social Isolation	−0.23	0.03	<0.01	0.39
White/European Ethnicity	−0.10	0.52	<0.01	0.01
Worry about COVID-19 impact on mental health	−0.13	0.24	<0.01	0.04

Adjusted R^2^ = 0.47. B = standardized Beta. Note: Cohen’s f2 was calculated for interpreting the effect size of each variable. f2 ≥ 0.02, f2 ≥ 0.15, and f2 ≥ 0.35 represent small, medium, and large effect sizes, respectively [31,32].

## Data Availability

The data presented in this study are available on request from the corresponding author. The data are not publicly available due to privacy.

## Supplementary Materials

The following are available online at https://www.mdpi.com/article/10.3390/ijerph18136730/s1, Table S1: LASSO Model for Psychological Wellbeing, Table S2: Summary of COVID-Specific Items.
