# Supplementary material for: Identifying Predictors of University Students’ Wellbeing during the COVID-19 Pandemic—A Data-Driven Approach
Source: Int J Environ Res Public Health. 2021 Jun 22;18(13):6730. doi: 10.3390/ijerph18136730 (PMC8296899; doi:10.3390/ijerph18136730)
Supplement: Supplementary file 1 [file ijerph-18-06730-s001.zip › ijerph-1229494-supplementary.pdf]

Data Availability Statement: The data presented in this study are available on request from the corresponding author. The data are not publicly available due to privacy.

Table S1: LASSO Model for Psychological Wellbeing

Variables	Coefficient
Age	0
Female Sex	0
International Students	0
Enrolled in Australia	0
Level of Education	
Undergraduate	0
Undergraduate with Double Degree	0
Honour	0
Postgraduate	0
Ethnicity	
East Asian	0
South Asian	0
Southeast Asian	0
White/European	0
Other	0

Living Conditions	
Live Alone	0
Live on campus	0
Time Point	
May	0
July	0
August	0
October	0
December	0
**Physical Health Status**	3.76
COVID-related Items	
Worry about being infected	0
Worry about friends or family being infected	0
Worry about physical health being influenced by Coronavirus	0
**Worry about mental health being influenced by Coronavirus**	-2.07
Worry about having enough money and resources	0

Worry about staying safe when leaving the house	0
**Restriction Stress**	-1.02
Lifestyle Factors	
Time spent outside during last two weeks	
No days	0
1-2 days per week	0
3-4 days per week	0
5-6 days per week	0
Everyday	0
**Diet Worsened**	-0.55
**Perceived Sufficiency of Distance Communication**	-0.45
**Psychological Factors**	
**Emotional Support**	0.28
**Social Isolation**	-0.63
**Resilience**	0.82

Root Mean Square Error (RMSE): 14.85

Table S2: Summary of COVID-Specific Items

**Item**
During the last two weeks, have you been worried about… (responses based on 5-Point Likert Scale)
being infected?
friends or family being infected?
your physical health being influenced by Coronavirus/COVID-19?
having enough money and resources?
staying safe when you leave the house?
During the last two weeks, how stressful have the restrictions on leaving home been for you (responses based on 5-Point Likert Scale)?
